# Effectiveness of Telerehabilitation Interventions for Self-Management of Tinnitus: Update of a Systematic Review

**DOI:** 10.2196/83529

**Published:** 2026-02-27

**Authors:** Sara Demoen, Elise Van Kerchove, Annick Timmermans, Vincent Van Rompaey, Sarah Michiels, Annick Gilles

**Affiliations:** 1 REVAL Rehabilitation Research Center Faculty of Rehabilitation Sciences Hasselt University Hasselt Belgium; 2 Department of Translational Neurosciences Faculty of Medicine and Health Sciences University of Antwerp Antwerp, Flanders Belgium; 3 Department of Otorhinolaryngology and Head and Neck Surgery Faculty of Medicine and Health Sciences Antwerp University hospital Antwerp Belgium; 4 Rehabilitation Research Group (RERE) Faculty of Health Sciences Vrije Universiteit Brussel Brussels, Brussels Capital Belgium; 5 Department of Education, Humanities and Social Work Faculty of Human and Social Welfare University College Ghent Ghent, Flanders Belgium

**Keywords:** tinnitus, telerehabilitation, self-management, mobile phone, smartphone application

## Abstract

**Background:**

Approximately 14% of the adult population has tinnitus, and current treatments are often costly and time-consuming. Telerehabilitation might reduce treatment costs without compromising effectiveness.

**Objective:**

Telerehabilitation is a quickly evolving research topic. Therefore, this systematic review update aims to give an overview of the research concerning the effectiveness of telerehabilitation interventions for self-management of tinnitus published between 2022 and 2025.

**Methods:**

This systematic review adheres to the PRISMA (Preferred Reporting Items for Systematic Reviews and Meta-Analyses 2020) guidelines. PubMed, ScienceDirect, Scopus, Web of Science, and Cochrane Library were consulted for eligible studies concerning a study intervention of any possible form of self-management or telerehabilitation for adult patients with subjective tinnitus as a primary complaint. The risk of bias (RoB) and certainty of all included studies were assessed respectively by the Cochrane RoB2-tool and GRADE (Grading of Recommendations, Assessment, Development, and Evaluation) framework.

**Results:**

In total, 24 papers were included, of which 6 studied multiple telerehabilitation forms. Internet-based cognitive behavioral therapy with guidance by a psychologist or audiologist was examined in 5 studies (n=619), self-help manuals in 1 study (n=10), technological self-help devices in 3 studies (n=286), smartphone apps in 13 studies (n=23,788), and other internet-based interventions in 5 studies (n=442). These rehabilitation categories were proven to be effective in decreasing tinnitus severity and relieving tinnitus distress as measured by tinnitus questionnaires.

**Conclusions:**

The strength of this review is the gathering of recent studies on the very evolving topic of telerehabilitation for tinnitus. An important limitation of all included studies is that they raised some to great concerns of RoB. As a result, it is necessary to acknowledge that the overall certainty of the evidence ranged from low to moderate certainty. In addition, some crucial confounding parameters, such as the presence of hearing loss, hyperacusis, anxiety, depression, or sleeping problems, were not taken into consideration by all studies. This review gives an indication of the use of different telerehabilitation and self-management interventions for real-world clinical use, stating not only their possibilities but also their limitations. Overall, telerehabilitation was found to be effective in reducing tinnitus severity and distress. It forms a possible tool to improve the self-management capacities of the patient and the accessibility of tinnitus care as a replacement or an addition to in-person care. Nevertheless, barriers such as a lack of time, engagement, motivation, and openness of the patient, causing high dropout, should be taken into consideration. This review accentuated the shift from internet-based cognitive behavioral therapy to the growing interest in the use of smartphone apps, increasing the accessibility of the treatments even more.

**Trial Registration:**

PROSPERO CRD 42021285450; https://www.crd.york.ac.uk/PROSPERO/view/CRD42021285450

## Introduction

### Background

Tinnitus is defined as the perception of a constant or intermittent sound without the presence of an external auditory source [1,2]. It is a highly prevalent symptom affecting approximately 14% of the adult population [3]. The daily functioning and health-related quality of life of tinnitus patients can directly or indirectly be disrupted by the distress they experience due to the tinnitus sound [4]. Therefore, the most commonly used and evidence-based treatments are tinnitus retraining therapy (TRT) and cognitive behavioral therapy (CBT) [5,6]. The high costs and time consumption of these treatments, combined with the repeated need for care of these patients, resulted in a quest to find a more cost-effective alternative [7-9]. As an answer, telerehabilitation is on the rise to promote self-management in tinnitus patients and reduce their dependency on in-person care. Telerehabilitation, meaning delivery of care from a distance, became a necessity rather than an opportunity during the COVID-19 pandemic [10-12].

The previous systematic review published in 2023 on the topic of telerehabilitation for chronic subjective tinnitus patients indicated that low-contact treatment provided from a distance through several telerehabilitation forms could be used as a substitution for or an addition to in-person clinical care for tinnitus [13]. It can potentially reduce the costs of treatment without sacrificing the level of effectiveness. Telerehabilitation treatment forms are easy to implement in clinical practice and form accessible treatment options [13]. Overall, telerehabilitation in the form of internet-based cognitive behavioral therapy (iCBT) with or without guidance, self-help manuals, self-help devices, smartphone apps, and other internet-based interventions was found to be effective in reducing tinnitus severity and distress [13]. Nevertheless, barriers such as lack of time, engagement, motivation, and openness of the patient need to be considered, as they can cause high dropout rates. The previous review also concluded that future research should further explore which factors are most likely to cause the lack of compliance and how clinicians can counteract these [13].

Due to the advances in technology made every day, telerehabilitation will keep evolving and, therefore, remain a trending topic to follow up on. The application of digital technology in tinnitus therapy is rising stronger than ever. For example, a variety of smartphone apps for people with tinnitus are available, promising relief from the condition. Yet, it is difficult to estimate their potential [14,15]. After the COVID-19 pandemic, the potential benefits of telerehabilitation forms became undeniable, and recently, these new tinnitus treatment forms were even implemented in the guidelines for tinnitus care. Since then, telehealth has been a highly dynamic research field, and several new studies on telerehabilitation for tinnitus have been published in the last couple of years [16-18]. The high number of patients with tinnitus, their repeated need for care, long waiting lists for regular care, and the high costs related to gold-standard treatments are some of the reasons why the need to explore telerehabilitation options is so high. It might form the key to making treatments more cost-effective and more accessible without losing on a level of effectiveness. It can even benefit the effectiveness when it is used as a tool to improve tailoring of the treatment approach to the patient’s needs and as a tool to increase adherence and self-management skills of the patient, reducing the need for repeated care.

### Objectives

In the past decades, various low-contact and self-management telerehabilitation treatments were developed for patients with tinnitus to search for an alternative or additional tool for the existing treatment options that are time-consuming and costly. Due to the rise in scientific research and the high number of new publications in recent years on this topic, this systematic review aimed to give an updated overview of the research concerning the effectiveness of telerehabilitation interventions for self-management of tinnitus published between 2022 and 2025.

## Methods

### Study Design

This systematic review is an update of an earlier systematic review published in February 2023 [13]. It adheres to the PRISMA (Preferred Reporting Items for Systematic Reviews and Meta-Analyses) guidelines [19]. The predefined protocol registered in PROSPERO from the earlier review, with registration number CRD42021285450, was repeated identically.

### Eligibility Criteria

The eligibility criteria can be found in Textbox 1. These criteria remained the same for the updated review. However, a publication date restriction was added as an exclusion criterion. Papers published before October 18, 2021, were not taken into consideration because they were already included in the previous review. The search ended on February 10, 2025.

**Eligibility criteria.**

**Inclusion criteria**

- Adult patients (>18 y).
- Subjective tinnitus as a primary complaint.
- A study intervention comprising any possible form of self-management or telerehabilitation.
- No publication date restrictions.

**Exclusion criteria**

- No tinnitus.
- Objective tinnitus.
- Tinnitus as a secondary complaint.
- Full text not available in Dutch, French, German, Greek, or English.

### Information Sources

On February 10, 2025, a total of 5 databases were consulted: PubMed, ScienceDirect, Scopus, Web of Science, and Cochrane Library.

### Search Strategy

The search queries were composed according to the PRISMA-S (Preferred Reporting Items for Systematic Reviews and Meta-Analyses Literature Search Extension) guidelines [20] (checklist provided in Multimedia Appendix 1). The queries comprise free terms and MeSH (Medical Subject Headings) terms for tinnitus (population) and self-management, telerehabilitation, smartphone application (intervention), and their synonyms. All terms and synonyms were controlled for value to the search query. If a term did not result in an additional number of studies found, it was eliminated to keep the search query as concise as possible while maintaining the highest level of efficiency. The search queries were not adapted from the previous review and were rerun identically, because the addition of terms did not result in more relevant eligible studies. The complete search queries can be consulted in Multimedia Appendix 2. All searches were concluded on February 10, 2025. Besides the date restriction that papers had to be from 2022 or later, no additional search filters or restrictions were added to the search strategy. Rayyan was used to eliminate duplicates from the searches.

### Selection Process

Double screening of the gathered papers was performed by 2 reviewers (Sara Demoen and EVK) independently and blindly. A first screening on the abstract and title was performed, followed by a screening on the full text. The Rayyan web platform was used to screen all papers efficiently. The final decision on the inclusion of the papers was obtained after a consensus meeting, discussing potential conflicts or papers that were labelled as maybe by at least one of the reviewers.

### Data Collection

Both reviewers collected the data independently, and the data extraction tables were composed during a consensus meeting with the reviewers and the supervisors. In the earlier review, 6 subcategories were made: iCBT with guidance (feedback, monitoring, and support by a psychologist or audiologist), iCBT without guidance, self-help devices, self-help manuals, smartphone apps, and other internet-based interventions. For all of these categories, new studies were included during this updated search, except for the subcategory iCBT without guidance. For each subcategory, a data-extraction table was composed, comprising the collected study information according to the applicable items of the subdivisions: PICO (population, intervention, comparison, and outcome).

### Data Items

The study and population characteristics that were listed are the author, the year of publication, the study design, the sample size (n), the gender distribution (%), the mean age and the SD (y), the type of tinnitus, the mean duration of tinnitus, and the presence of hearing loss.

Intervention is the first aspect summarized in the table of evidence. This section discusses the following items: (+ if applicable comparison: control intervention, ie, if there is a comparison with a control group [CG], these items are also discussed for the control intervention and not solely the experimental intervention): the used intervention form and specifics, such as guidance, duration, and follow-up. For the next section, Outcome, outcome measures, and dropout were presented, and finally, under the Results section, data results, GRADE (Grading of Recommendations, Assessment, Development, and Evaluation) score, and the conclusions were listed. Each of the 5 subcategories of telerehabilitation included in this review had a table of evidence giving an overview of the information on the intervention, outcome, results, and conclusions of the included study.

### Risk of Bias Assessment

The risk of bias (RoB) assessment was performed using the Cochrane RoB2 tool, a tool for assessing RoB in randomized controlled trials (RCTs) [21]. However, it can also be used for other study designs because the domains are, in general, also applicable [21]. The study design of the included studies is diverse, and for this reason, following a separate RoB assessment for each study design would not give a clear overview for comparison [21]. Alternative tools such as ROBINS-I (Risk of Bias in Non-Randomized Studies of Interventions) were considered, but using a different instrument in the current update would introduce heterogeneity in assessment methods. We therefore applied the Cochrane RoB tool across all designs to ensure methodological consistency with the previous version of this review, and because this tool provides a more conservative, strict assessment of bias for nonrandomized studies rather than an overly lenient one. RoBvis, an RoB visualization tool by Cochrane, was used to summarize the results in a graphic manner [22]. The RoB screening was performed blinded by 2 reviewers (Sara Demoen and EVK), and conflicts were resolved after a consensus meeting.

### Data Synthesis

The heterogeneity in reported interventions and outcome measures precluded meta-analysis. A meta-analysis was not performed in the present review update for several methodological reasons. First, this work serves as an update of a previously published systematic review, which also did not pool results due to substantial heterogeneity. Maintaining consistency with the earlier review ensures comparability across iterations. Second, the included studies used a wide range of outcome measures that were operationalized and reported in noncomparable ways, limiting the feasibility of quantitative synthesis. Finally, the statistical reporting across studies varied considerably, with insufficiently detailed or inconsistent data formats that prevented reliable extraction of effect sizes. In combination, these factors precluded a valid and meaningful meta-analysis, and therefore, it was decided to summarize the collected data in a systematic review. This was not different in the current update.

### Certainty Assessment

The GRADE framework is a tool to assess the level of certainty by examining RoB, imprecision, inconsistency, indirectness, and publication bias of the included studies [23]. The GRADE assessment was completed according to the GRADE handbook guidelines [23].

## Results

### Study Selection

**Figure 1:**
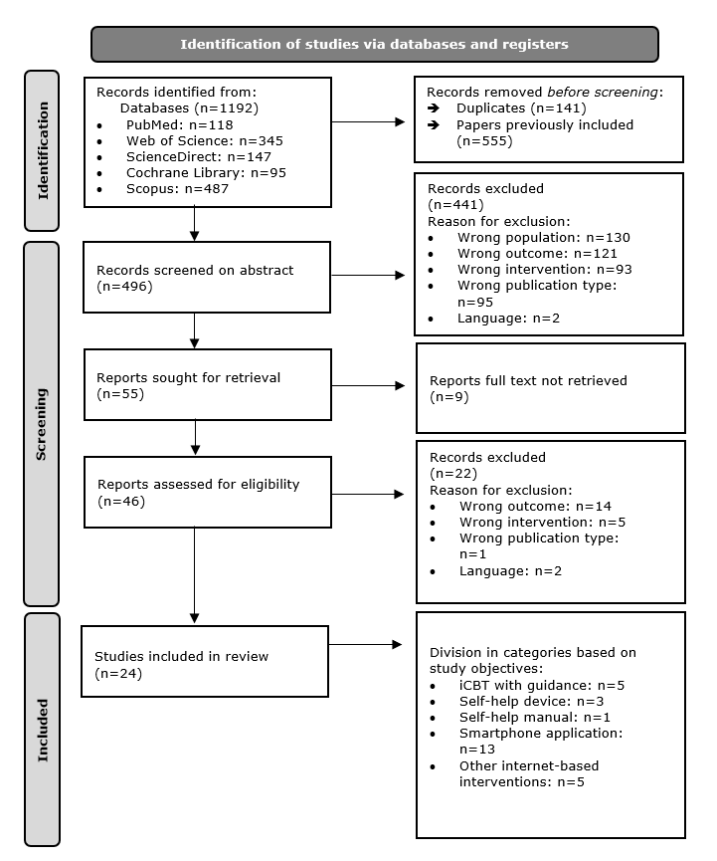
Flow diagram for study inclusion (PRISMA 2020). Studies can belong to multiple of the listed study objective categories. iCBT: internet-based cognitive behavioral therapy; PRISMA: Preferred Reporting Items for Systematic Reviews and Meta-Analyses. S

The initial database searches on February 10, 2025, resulted in 1192 potentially relevant results (Figure 1 [19]). After removal of the duplicates and of the papers published before October 2021, a total of 496 papers remained for primary screening on title and abstract. During the primary screening, 441 papers were excluded; the reasons for exclusion are specified in Figure 1. In total, 55 papers were sought for retrieval for the screening of full text. Subsequently, the screening of the full text was completed for 46 papers because the full text of 9 papers could not be retrieved. The reason for exclusion is again specified in Figure 1. A total of 24 papers were found to be eligible [24-47]. These eligible papers were divided into the same subgroups used in the previous systematic review [13]. The subgroups were based on the form of telerehabilitation as an intervention: iCBT with guidance, iCBT without guidance, self-help devices, self-help manuals, smartphone apps, and other internet-based interventions.

### About RoB

#### Controlled Trials

**Figure 2:**
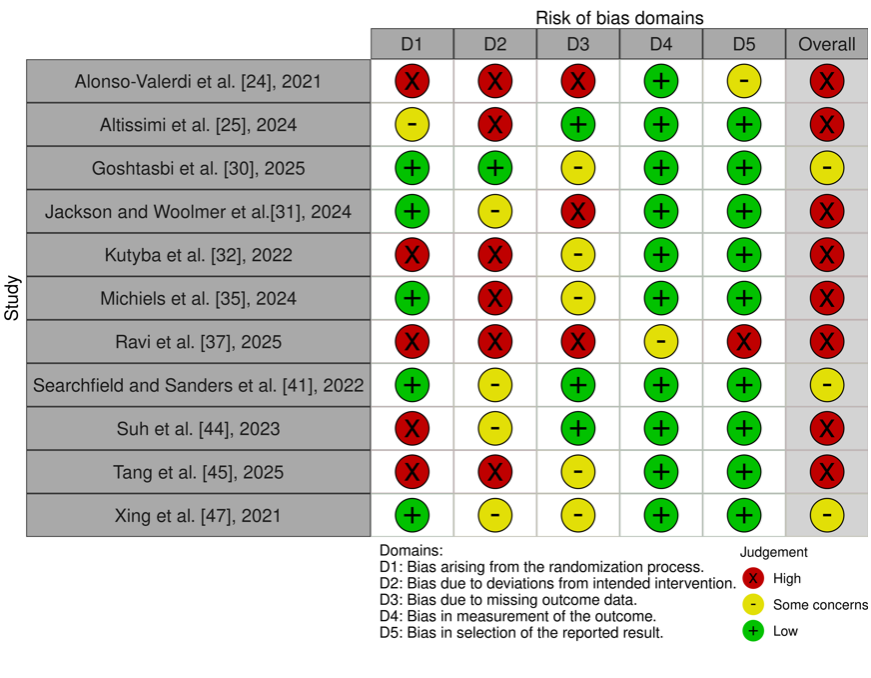
Risk of bias in the controlled trials scoring per domain [25,26,31,32,33,36,38,42,45,46,48].

In total, 11 studies were controlled trials [24,25,30-32,35,37,41,44,45,47], of which 6 studies were RCTs [25,30,31,35,41,47]. Five RCTs had a low RoB arising from the randomization process, except for Altissimi et al [25], who showed a higher RoB due to the lack of information given concerning the randomization method (D1) [25,30,31,35,41,47]. All controlled trials showed some reasons for concern of RoB due to deviations from the intended interventions (D2), except for Goshtasbi et al [30]. RoB, due to missing outcome data (D3), was scored as “high concerns” and “some concerns” for studies with respectively moderate to high dropout rates or no clearly specified plan on how to handle these missing data. All controlled studies were rated “low concerns for RoB” in the measurement of outcome (D4), except Ravi et al [37], a study with a sample size of only 2 patients and only 1 questionnaire as an outcome measure. Most studies had a full, clear, correct, and specified plan for statistical analysis, meaning the concern for RoB in the selection of the reported result (D5) was low. However, Alonso-Valerdi et al [24] and Ravi et al [37] had a nondetailed protocol, missing information on the exact premised data analysis plan. Overall, studies were rated “some concerns” to “high concerns” of RoB. See Figure 2 [24,25,30-32,35,37,41,44,45,47] for the RoB assessment results of the controlled trials. Additionally, a summarization of RoB results can be found in Multimedia Appendix 3.

#### Noncontrolled Trials

**Figure 3:**
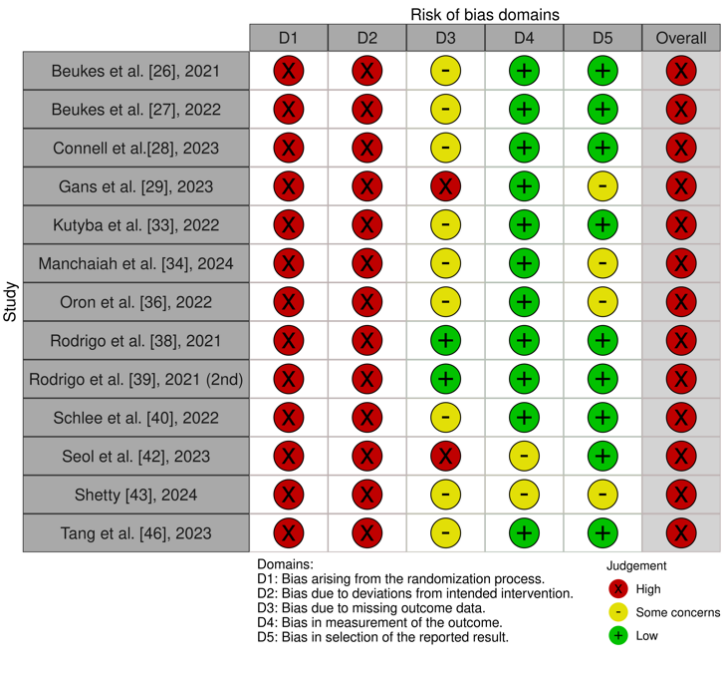
Risk of Bias in the noncontrolled trials per domain [27,28,29,30,33,35,37,39,40,41,43,44,47].

A total of 13 studies were noncontrolled trials [26-29,33,34,38-40,42,43,46]. They all show some concerns about RoB due to a lack of randomization, blinding, and CGs (D1 and D2). RoB due to missing outcome data (D3) was again scored high, and there were some concerns for studies with moderate to high dropout rates. The majority of the studies specified their statistical analysis beforehand, resulting in low RoB in the selection of the reported results (D5). See Figure 3 [26-29,32,34,36,38-40,42,43,46] for the RoB assessment results of the noncontrolled trials.

### Certainty

In general, the included studies had a low to moderate certainty level according to the GRADE framework [23].

### iCBT with Guidance

#### Study and Patient Characteristics

A total of 3 studies looked into the use of an iCBT program with guidance provided by an audiologist in the form of feedback, monitoring, and support through an online messaging system within the platform [26,27,34]. All 3 studies were cohort studies with an adult study population with chronic tinnitus. Further, 2 studies additionally specified that tinnitus symptoms had to be present for more than 3 months, and it had to be at least a mild severity of tinnitus. Beukes et al [26] stated that patients were only eligible if they scored at least 25 of 100 on the Tinnitus Functional Index (TFI). The mean tinnitus duration ranged from 11.64 to 13.91 years between the studies. Beukes et al [26] was the only study controlling for the presence of hearing loss, while Beukes et al [27] was the only one specifying the tinnitus characteristics, namely the tinnitus location. The total sample is 391 patients receiving iCBT with guidance. For further specifications, see Table S1 in Multimedia Appendix 4. Two studies examined an iCBT program with minimal guidance [38,39]. However, it needs to be noted that both these published studies were from the same author and concerned the same sample data. Both studies used data from a cohort of 228 patients with long-standing chronic tinnitus with a duration of 17.68 (SD 19.42). For further specifications, see Table S1 in Multimedia Appendix 4.

#### Results of Individual Studies

All 3 studies used a similar iCBT program consisting of an interactive e-learning program with 22 modules based on the original CBT self-help program developed by Andersson and Kaldo [48] (2004) [26,27,34]. The interventions were guided by an audiologist who monitored progress, provided feedback on worksheets that were completed, outlined the content of new modules, and answered questions. The guidance by the audiologist was offered through a 2-way encrypted online messaging system. The primary tinnitus outcome of all studies was tinnitus severity or distress measured by the TFI. Various secondary outcome measures, using self-report questionnaires, were appended. All studies used data from previous trials. See Table 1 for further details. All studies concluded that the iCBT with guidance provided by an audiologist trained in iCBT principles was effective in the treatment of chronic tinnitus. Beukes et al [26] demonstrated that iCBT has long-term positive effects, and Manchaiah et al [34] added that it can help individuals with tinnitus to think more positively by changing unhelpful patterns. Both studies from Rodrigo et al [38,39] used the same data that was gathered from multiple trials and looked into the use of an iCBT program with minimal guidance through an encrypted messaging system solely for support. This iCBT program was again based on the original CBT self-help program developed by Andersson and Kaldo [48] (2004). However, it was adapted to a set of only 21 modules, of which 5 were optional. These 2 studies also found a decrease in TFI after completing the iCBT program and additionally investigated predictors of iCBT outcomes. For more information, see Table 1.

**Table 1:** Table of evidence for the studies concerning iCBT^a^ with guidance.

Study	Intervention			Outcome			Results				
Author and year	Intervention	Treatment period	Follow-up	Primary tinnitus outcome measure	Additional outcome measures	Statistical analysis method	Data results	Statistical analysis results	Dropout	Conclusion	GRADE^b^
Beukes et al [26], 2022	Interactive e-learning iCBT consisting of 22 modules and guidance by an audiologist	8 weeks	FU1^c^: postintervention assessment; FU2^d^: 2-months postintervention; FU3^e^:1-year postintervention	TFI^f^	THS^g^.	Effect sizes, LMM^h^, and the RCI^i^.	Significant BL^j^-FU3 TFI mean improvement of 24.3 (SD 22.5; T0: 54.23, SD 20.7; T3: 30.15, SD 24.51). There was a clinically significant change for 56 (42%) of the participants using the reliable change criterion of 26.98 at T3.	Statistically significant BL-FU3 difference with a large effect size (Cohen *d*=1.06, CI 0.80 to 1.32).	68/200 (34%) did not complete FU1, 83/200 (43%) FU2, and 68/200 (34%) FU3.	The study provides evidence of long-term efficacy for an audiologist-guided iCBT tinnitus intervention.	⊕⊕⊕○
Beukes et al [27], 2022	Interactive e-learning iCBT consisting of 22 modules and guidance by an audiologist	8 weeks	FU^k^=2 months	TFI	GAD-7^l^, PHQ-9^m^, ISI^n^, EQ-5D-5L, THS, TCQ^o^, tinnitus and hearing survey.	Pretest and posttest effect size for all primary and secondary outcomes; 2-sided *P* values using α=.05.	A significant, large effect size TFI change postintervention (mean 1.60, 0.91-2.23), which was maintained at FU.	The RCI indicated a prepost score difference of 19.51 on the TFI would be a clinically significant change.	4/27 did not complete FU1 and 9/27 did not complete FU2.	This pilot study has indicated the feasibility of iCBT for tinnitus in the United States.	⊕⊕⊕○
Manchaiah et al [34], 2024	iCBT with guidance by an audiologist	8 weeks	FU: posttreatment	TFI	GAD-7, PHQ-9, ISI, EQ-5D-5L.	Mixed method approach, paired sample *t* test as the data met the assumption of normality using the Shapiro-Wilk test (*P*>.05).	More participants reported positive experiences following the intervention in the overall sample (preintervention mean 0.78, postintervention mean 1.63, *P*<.001).	53/164 (32.3%) provided at least 1 positive experience in both pre and postintervention (group 1), 19/164 (9.1%) provided positive experiences only during preintervention, 49/164 (29.9%) provided positive experiences only during postintervention, and 28.7 participants did not provide any positive experience in both pre- and postintervention.	NR^p^.	iCBT can help individuals with tinnitus to think more positively by changing unhelpful thought patterns.	⊕⊕⊕○
Rodrigo et al [38], 2022	Interactive e-learning iCBT consisting of 21 modules and minimal guidance by an audiologist	8 weeks (2-3 modules a week)	FU1=postintervention assessment; FU2=2-months postintervention	TFI	ISI, PHQ^q^, HHIA-S^r^, HQ^s^, CFQ^t^, SWLS^u^.	Paired *t* test.	The mean BL tinnitus severity and tinnitus severity following the iCBT intervention were 57.93 (SD 19.17) and 34.22 (SD 22.76), respectively, indicating a significant decrease (*P*<.001).	65% had a successful iCBT outcome, meaning they had a 13-point or higher reduction in TFI after the intervention.	NR.	Some predictors can be noted for a better outcome of iCBT, such as, for example, level of education, BL tinnitus, workload, and disability allowance.	⊕⊕⊕○
Rodrigo et al [39], 2021	iCBT	8 weeks (2-3 modules a week)	FU: postintervention	TFI	ISI, PHQ, HHIA-S, HQ, CFQ, SWLS.	Paired sample *t* tests.	Undertaking iCBT significantly reduced tinnitus severity scores (t_227_=16.37; *P*<.001) from a mean BL severity score of 57.93 (SD 19.17) to a mean post-iCBT severity score of 34.22 (SD 22.78), as measured by the TFI.	A clinically significant 13-point change in TFI scores was achieved by 150 of the 228 participants after the intervention.	NR.	Decision tree models, especially the CART^v^ and gradient boosting models, appeared to be promising in predicting iCBT outcomes.	⊕⊕⊕○

^a^iCBT: internet-based cognitive behavioral therapy.

^b^GRADE: Grading of Recommendations, Assessment, Development, and Evaluation.

^c^FU1: first follow-up moment.

^d^FU2: second follow-up moment.

^e^FU3: third follow-up moment.

^f^TFI: tinnitus functional index.

^g^THS: tinnitus handicap scale.

^h^LMM: linear mixed effects models.

^i^RCI: Reliable Change Index.

^j^BL: baseline.

^k^FU: follow-up moment.

^l^GAD-7: Generalized Anxiety Disorder-7.

^m^PHQ-9: Patient Health Questionnaire-9.

^n^ISI: Insomnia Severity Index.

^o^TCQ: Tinnitus Cognitions Questionnaire.

^p^NR: not reported.

^q^PHQ: Physical Health Questionnaire.

^r^HHIA-S: Hearing Handicap Inventory for Adults-Short Form.

^s^HQ: Hyperacusis Questionnaire.

^t^CFQ: Cognitive Failures Questionnaire.

^u^SWLS: Satisfaction With Life Scale.

^v^CART: Communication Access Realtime Translation.

### Self-Help Devices

#### Study and Patient Characteristics

Three controlled trials, of which 2 RCTs, studied the use of self-help devices for the treatment of chronic tinnitus [24,25,41]. The studies had a total sample size of 286 patients. The specifications of all 3 studies can be consulted in Table S2 in Multimedia Appendix 4.

#### Results of Individual Studies

All 3 studies looked into the use of a self-help device delivering a form of sound therapy. For Altissimi et al [25], this was the use of a sound generator for TRT treatment, and this was the CG. The intervention group received TRT treatment using a smartphone app. Searchfield and Sanders [41] also used a smartphone app in combination with the self-help devices for sound therapy, and the CG did as well use an app, but without a self-help device. All studies concluded that the use of a self-help device was effective in reducing tinnitus distress according to the TFI or Tinnitus Handicap Inventory [24,25,41]. Further details can be found in Table 2.

**Table 2:** Table of evidence for the studies concerning self-help devices.

Study	Intervention					Outcome				Results				
Author and year	Intervention	Control group	Treatment period	Follow-up	Primary tinnitus outcome measure		Additional outcome measures	Statistical analysis method	Data results		Statistical analysis results	Dropout	Conclusion	GRADE^a^
Alonso-Valerdi et al [24], 2021	(1) TRT^b^; (2) TEAE^c^; (3) ADT^d^; (4) BBT^e^; (5) music	No treatment.	2 months (60 min daily)	FU1^f^: after 1 week; FU2^g^: after 5 weeks; FU3^h^: after 8 weeks	THI^i^		HADS^j^, audiometry, tinnitus characteristics analysis, and BL^k^ neurophysiological analysis	Identification of ± or 0 effects *t* test	All negative differences between BL and FU^l^ were statistically significant (*P*=.009)		29.4% of the TRT group reported diminution of their tinnitus perception, followed by those in TEAE (18.8%), BBT or ADT (14.3%), and music (7.7%) groups.	Dropout: music 6/16, BBT^m^ 4/18, TRT 3/18, TEAE 5/18, ADT 5/18, and control 3/14	TRT most effective sound-based therapy to reduce tinnitus perception for individuals without anxiety. BBT, ADT, and TEAE produced very similar effects.	⊕⊕⊕○
Altissimi et al [25], 2024	TRT with mobile app	TRT with a traditional sound generator.	6 months	FU1: after 3 months of treatment; FU2: after 6 months of treatment	THI		TRT interview: with questions on tinnitus, hearing loss, hyperacusis, classifying gravity, the annoyance, and the effect on the QoL^n^ of tinnitus, using a VAS^o^.	Descriptive data by counts of a mixed-effect model Tukey test	A significant difference in the THI scores was found in category 1 patients between those treated with a sound generator vs app (–14.529, *P*<.001)		Improvement was found in category 0 patients, both treated with the app and sound generator; conversely, in category 1, significant improvement was seen only for the group using a sound generator.	No dropout	TRT can be delivered to patients with mild tinnitus (class 0) through mobile apps with results comparable to traditional sound generators.	⊕⊕⊕○
Searchfield and Sanders [41], 2022	The digital therapeutic consisted of an app for iPhone or Android smartphone, Bluetooth bone conduction headphones, a neck pillow speaker, and a cloud-based clinician dashboard to enable messaging and app personalization	The control app was a popular self-help passive sound therapy app called WN^p^ Lite.	12 weeks	FU1: midtrial=6 weeks; FU2: postinterevention	TFI^q^		COSIT^r^, SUS^s^, MAUQ^t^	ANOVA with 2 groups and 4 repeated measures, intent-to-treat and completer (per protocol) analyses	Mean changes TFI for USL^u^ at 6 (16.36, SD 17.96 points) and 12 weeks (17.83, SD 19.87 points) were clinically meaningful (>13 reduction), the mean changes in WN group were not (6 wk 10.77, SD 18.53 points; 12 wk 10.12, SD 21.36 points)		A statistically higher proportion of USL participants achieved meaningful TFI change at 6 weeks (55%) and 12 weeks (65%) than the WN group at 6 weeks (33%) and 12 weeks (43%).	4/35 USL and 6/36 WN	The USL group demonstrated a higher proportion of responders than the WN group. The usability of the USL therapeutic was similar to the established WN app. The digital polytherapeutic demonstrated significant benefit for tinnitus reduction, supporting further development.	⊕⊕⊕○

^a^GRADE: Grading of Recommendations, Assessment, Development, and Evaluation.

^b^TRT: tinnitus retraining therapy.

^c^TEAE: therapy for an enriched acoustic environment.

^d^ADT: auditory discrimination therapy.

^e^BBT: broad band therapy.

^f^FU1: first follow-up moment.

^g^FU2: second follow-up moment.

^h^FU3: third follow-up moment.

^i^THI: Tinnitus Handicap Inventory.

^j^HADS: Hospital Anxiety and Depression Scale.

^k^BL: baseline.

^l^FU: follow-up moment.

^m^BBT: Broad band therapy.

^n^QoL: quality of life.

^o^VAS: visual analog scale.

^p^WN: white noise.

^q^TFI: Tinnitus Functional Index.

^r^COSIT: Client-Oriented Scale of Improvement in Tinnitus.

^s^SUS: System Usability Scale.

^t^MAUQ: mHealth App Usability Questionnaire.

^u^USL: UpSilent app.

### Self-Help Manual

#### Study and Patient Characteristics

Only 1 cohort with a sample size of 10 adult patients with tinnitus and with bilateral mild sloping sensorineural hearing loss who wore a hearing aid used a self-help manual as an intervention [43]. Further details can be found in Table S3 in Multimedia Appendix 4.

#### Results of Individual Studies

Shetty [43] examined the use of a self-management manual for tinnitus with content such as (1) hearing aids; (2) listening to songs or soothing sounds; (3) tips for food intake; (4) exercise, yoga, and meditation; (5) locking negative thinking; (6) distraction techniques; (7) progressive muscle relaxation; (8) slow breathing; and (9) attention control and imaginary techniques. The self-help manual was prepared using the health literacy recommendations by adapting the United States Department of Health and Human Services. It was concluded that the self-management manual effectively reduces tinnitus perception and associated problems [43]. See Table 3 for further information.

**Table 3:** Table of evidence for the studies concerning the self-help manual.

Study	Intervention					Outcome				Results				
Author and year	Intervention	Control group	Treatment period	Follow-up	Primary tinnitus outcome measure		Additional outcome measures	Statistical analysis method	Data results		Statistical analysis results	Dropout	Conclusion	GRADE^a^
Shetty et al [43], 2024	Self-management manual for tinnitus	N/A^b^	30 days	FU^c^: postintervention	THI^d^, TFI^e^		N/A	Independent samples *t* test.	The results revealed a significant reduction in THI after 30 days of manual use (*t*_9_=1.99, *P*=.07). A similar result was found in TFI, in which functional impairment from tinnitus was significantly reduced between visits one and two (*t*_9_=6.13, *P*=.001)		The means of THI and TFI scores in visit 1 were 31 (SD 5.16) and 58 (SD 5.92), respectively. Furthermore, at visit 2, the THI and TFI scores were 29 (SD 4.59) and 45 (SD 7.05), respectively.	No dropout	Patients who have used the manual reported relaxing and coping with tinnitus. It is concluded that the self-management manual effectively reduces tinnitus perception and associated problems.	⊕⊕⊕○

^a^GRADE: Grading of Recommendations, Assessment, Development, and Evaluation.

^b^N/A: not applicable.

^c^FU: follow-up moment.

^d^THI: Tinnitus Handicap Inventory.

^e^TFI: Tinnitus Functional Index.

### Smartphone Apps

#### Study and Patient Characteristics

A total of 13 articles, with a combined sample size of 23,637 participants, discussed the use of a smartphone app as part of the treatment of tinnitus patients [25,30,32,33,35-37,40-42,44-46]. Clarification on the study and patient characteristics of the studies examining the use of a smartphone app is noted in Table S4 in Multimedia Appendix 4.

#### Results of Individual Studies

The intervention used a smartphone app in 13 studies [25,30,32,33,35-37,40-42,44-46]. First, in 8 studies, this smartphone app was used to deliver a form of sound therapy to the patients. These studies showed that sound therapy through a smartphone app can be beneficial in reducing tinnitus distress. In 2 of these studies, Goshtasbi et al [30] and Searchfield and Sanders [41], the smartphone app combined both sound therapy and CBT or psychoeducation, respectively. Second, 5 studies look into TRT, CBT, or psychoeducational treatment delivered through a smartphone app. TRT with a mobile app appeared to be as effective as traditional TRT with or without a sound generator, and daily training using a CBT-based app targeting maladaptive beliefs may decrease tinnitus intrusiveness and handicap. Psycho-education through a smartphone app gave promising results in reducing tinnitus distress, but did not result in changes in tinnitus loudness. Finally, 1 study investigated the use of a smartphone app in the treatment of a specific subpopulation of tinnitus patients, namely patients with somatic tinnitus [35]. This pilot study provides evidence that an app-based physiotherapy intervention with a cervical spine exercise program could be an effective option for treating somatic tinnitus [35]. See Table 4 for an overview of the detailed information for all studies concerning treatments using smartphone apps.

**Table 4:** Table of evidence for the studies concerning smartphone apps.

Study	Intervention					Outcome				Results				
Author and year	Intervention	Control group	Treatment period	Follow-up	Primary tinnitus outcome measure		Additional outcome measures	Statistical analysis method	Data results		Statistical analysis results	Dropout	Conclusion	GRADE^a^
Altissimi et al [25], 2024	TRT^b^ with mobile app	TRT with a traditional sound generator	6 months	FU1^c^: after 3 months of treatment; FU2^d^: after 6 months of treatment	THI^e^		TRT interview: with questions on tinnitus, hearing loss, hyperacusis, classifying gravity, the annoyance, and the effect on the QoL^f^ of tinnitus, using a VAS^g^.	Descriptive data by counts, a mixed-effect model Tukey test.	A significant difference in the THI scores was found in category 1 patients between those treated with a sound generator vs app (–14.529, *P*<.001), while no significance was found in category 0 patients (–1.186, *P*=.78).		Improvement was found in category 0 patients, both treated with the app and sound generator; conversely, in category 1, significant improvement was seen only for the group using a sound generator.	No dropout.	TRT can be delivered to patients with mild tinnitus (class 0) through mobile apps with results comparable to traditional sound generators.	⊕⊕⊕○
Goshtasbi et al [30], 2025	Smartphone-based CBT^h^ and customized sound therapy.	Waitlisted	8 weeks (1 weekly CBT module and 2 h daily sound therapy)	Posttreatment	TFI^i^		PHQ-9^j^, GAD-7^k^, PSQI^l^, PSS^m^.	Independent sample *t* test and chi-square tests of independence.	Subjects in the IG^n^ showed greater improvements on their TFI (16.7, SD 14.9 versus 1.9, SD 10.8 (*P*<.001) and on 7 of 8 subscales (*P* <.001-.002).		18 (38.3%) IG patients showed a TFI improvement of 20 or more points posttreatment.	15 patients dropped out between week 2 and week 8 and were not included in the IG.	Smartphone-based CBT, providing CBT and customized sound therapy, was effective in reducing symptom severity and improving anxiety, sleep, and mood.	⊕⊕⊕○
Kutyba et al [32], 2022	Free ReSound Tinnitus Relief app	N/A^o^	6 months (≥30 min/day)	FU1: after 3 months of treatment FU2: after 6 months of treatment	THI, TFI		User survey.	Repeated measures ANOVA with Bonferroni correction.	THI: *F*_2,102_=62.4; *P*<.001; e2=0.550, TFI: *F*_2,102_=28.9; *P*<.001; e2=0.362.		In the study, the general severity decreased after the first 3 months (THI: –6.5 in mean score in comparison to BL^p^; TFI: –6.3 in mean score) and again in the following 3 months of using the application (THI: –19.4; TFI: –14.3).	NR.	The sound therapy through the use of a smartphone app appeared to be beneficial in reducing subjective tinnitus distress.	⊕⊕⊕○
Kutyba et al [33], 2022	Free ReSound Tinnitus Relief app	Participants not willing to participate in the intervention group	6 months (≥30 min/day)	FU1: after 3 months of treatment FU2: after 6 months of treatment	THI		VAS, user survey.	Independent (unpaired) *t* test or a chi-square test, and a mixed-design ANOVA with Bonferroni adjustment for multiple comparisons.	After 3 months of using the app, the THI global score significantly decreased (*P*<.001) in the study group, decreasing again at 6 months (*P*<.001).		A clinically important change (>20) in the THI was reported by 39% (17/44) of the study group and in none of the controls.	Dropout rate: 53% (50/94) in the study group and 55% (29/53) in the control group.	Use of an app that generates background sounds appears to be an effective way of reducing tinnitus severity.	⊕⊕⊕○
Michiels et al [35], 2024	App-based cervical spine exercise program	Waiting list	9 weeks	FU: posttreatment	THI miniTQ^q^		N/A.	Independent sample *t* test and chi-square tests of independence, and mixed model ANOVA.	Reduction in THI and miniTQ for the IG, not for CG^r^. Strong significant decrease in THI (from mean 51.33, SD 23.77 to mean 38.96, SD 21.66) and miniTQ (from mean 13.27, SD 5.81 to mean 9.14, SD 5.27).		Effect sizes for THI were *d*=1.71 (95% CI 0.85 to 2.56) and for miniTQ *d*=1.02 (95% CI 0.24 to 1.80).	CG: 3 dropouts; IG: 4 dropouts.	This pilot study provides evidence that an app-based physiotherapy intervention could be an effective option for treating somatic tinnitus.	⊕⊕⊕○
Oron et al [36], 2022	Brief cognitive behavioral training for tinnitus relief using a mobile GGTI^s^ app	N/A	16 days (3 min/day)	FU1: at level 24 half way through the app; FU2: level 48 final level of the app	H-THI^t^		VAS, user survey.	Repeated measures ANOVA of completers.	A significant linear trend was present, *F*_1,13_=14.5, *P*=.002, *d*=2.12. Oron et al [36] indicated 5 was a steady decrease in the H-THI score of 28.57, 22.14, and 22.0 for BL, FU1, and FU2, respectively.		50% of completers have shown reliable change (indicated by their RCI^u^).	12/26 (46%).	Several minutes a day of training using a CBT-based app targeting maladaptive beliefs may decrease tinnitus intrusiveness and handicap.	⊕⊕⊕○
Ravi et al [37], 2025	Mobile-based TRT	Conventional face-to-face TRT	3 months (2×/week)	FU1: after 1 month of treatment; FU2: after 2 months of treatment; FU3: after 3 months of treatment	THI		N/A.	N/A.	Patient A: BL=40, FU1=40; FU2=36; FU3=24. Patient B: BL=36, FU1=24; FU2=16; FU3=14.		N/A.	N/A.	Mobile-based TRT might be a good option for patients who cannot attend in-person sessions.	⊕⊕○○
Schlee et al [40], 2022	Educational smartphone app	N/A	4 months	FU^v^: postintervention	THI, TS^w^ NRS^x^		N/A.	Wilcoxon Signed Rank Test.	Statistical significance between the pre- and postintervention phase (W=345, *P*=.02).		Improvements on the tinnitus severity NRS reached an effect size of 0.408, while the improvements on the THI^y^ were much smaller with an effect size of 0.168.	26 of 52 (50%) participants dropped out.	Educational training on a smartphone app without the guidance of a medical person can result in small to medium improvements in tinnitus distress measures, but not for tinnitus loudness.	⊕⊕⊕○
Searchfield et al [41], 2022	The digital therapeutic consisted of an app for iPhone or Android smartphones, Bluetooth bone conduction headphones, a neck pillow speaker, and a cloud-based clinician dashboard to enable messaging and app personalization	The control app was a popular self-help passive sound therapy app called WN^z^ Lite.	12 weeks	FU1: midtrial=6 weeks FU2: postinterevention	TFI		COSIT^aa^, SUS^ab^, MAUQ^ac^	ANOVA with 2 groups and 4 repeated measures intent-to-treat and completer (per protocol) analyses	Mean changes in TFI for the USL^ad^ group at 6 (16.36, SD 17.96 points) and 12 weeks (17.83, SD 19.87 points) were clinically meaningful (>13 points reduction), the mean change in WN scores were not clinically meaningful (6 weeks 10.77, SD 18.53 points; 12 weeks 10.12, SD 21.36 points).		A statistically higher proportion of USL participants achieved meaningful TFI change at 6 (55%) weeks and 12 (65%) weeks than the WN group at 6 (33%) weeks and 12 (43%) weeks.	4/35 USL and 6/36 WN.	The USL group demonstrated a higher proportion of responders than the WN group. The usability of the USL therapeutic was similar to the established WN app. The digital polytherapeutic demonstrated significant benefit for tinnitus reduction supporting further development.	⊕⊕⊕○
Seol et al [42], 2023	Sound therapy smartphone app	N/A	6 months	FU1: midtrial=3 months; FU2: postinterevention	THI		BDI^ae^, SSL^af^, VAS stress assessment, hearing tests.	1-way RM^ag^ ANOVA as it passed the normality test (Shapiro-Wilk).	The THI scores, on the other hand, significantly decreased over the 6-month period (*P*=.02); when the Bonferroni correction method was applied for all *P* values, statistical significance was observed for THI scores between visits 1 and 3 (*P*=.006).		After 6 months of listening to the sound stimuli, participants’ perceived tinnitus handicap severity significantly decreased.	NR^ah^.	The findings of this study demonstrate the potential benefit of the tinnitus application for tinnitus improvement.	⊕⊕⊕○
Suh et al [44], 2023	Smart device–based TRT with smart pad app	Conventional TRT	3 months (3 sessions 30-60 min) with 1 month interval	FU1=postintervention assessment; FU2=2-months postintervention	THI		VAS, STAI^ai^, BDI, PSQI, pure tone audiometry.	*t* tests and chi-square tests.	The greatest THI improvement at 3 months was –23.3 (95% CI –33.41 to –13.4), and at 2 months –16.8 (95% CI –30.8 to –2.8), and the reductions were significant, *P*<.001.		There was no difference between IG and CG (*P*<.001).	NS^aj^.	TRT based on smart devices can be an effective alternative to conventional in-person TRT.	⊕⊕⊕○
Tang et al [46], 2022	FTRS^ak^ mobile app	Cohort	2 h/d	FU every 3 months	THI		HADS^al^, AIS^am^, VAS, FTQ^an^, TCS^ao^.	Descriptive statistics.	N/A.		N/A.	Only 22.9% of patients adhered to the application (29,864 patients were excluded because of incomplete records of personal and tinnitus-related information).	FTRS greatly reduced medical costs in comparison to traditional in-person care and enabled patients with tinnitus to arrange their own treatment times.	⊕⊕⊕○
Tang et al [45], 2025	DCRS^ap^ through a smartphone app	Unmodified music	3 months (2 h/d)	FU1: 1 month of treatment; FU2: 2 months of treatment; FU3: 3 months of treatment	THI		HADS, AIS, FTQ, TCS.	Linear mixed models and a multiclass logistic model with a stepwise function.	DCRS-treated patients after 3 months of treatment had significant tinnitus relief compared to those in the CG group (*P*<.001).		At 3 months, 92.5% of patients undergoing DCRS reported tinnitus relief or disappearance.	NS.	DCRS showed to be a promising noninvasive therapy for chronic tinnitus that can be delivered through a mobile app.	⊕⊕⊕○

^a^GRADE: Grading of Recommendations, Assessment, Development, and Evaluation.

^b^TRT: tinnitus retraining therapy.

^c^FU1: first follow-up moment.

^d^FU2: second follow-up moment.

^e^THI: Tinnitus Handicap Inventory.

^f^QoL: quality of life.

^g^VAS: visual analog scale.

^h^CBT: cognitive behavioral therapy.

^i^TFI: Tinnitus Functional Index.

^j^PHQ-9: Patient Health Questionnaire-9.

^k^GAD-7: Generalized Anxiety Disorder-7.

^l^PSQI: Pittsburgh Sleep Quality Index.

^m^PSS: Perceived Stress Scale.

^n^IG: intervention group.

^o^N/A: not applicable.

^p^BL: baseline.

^q^miniTQ: Mini Tinnitus Questionnaire.

^r^CG: control group.

^s^GGTI: GG Tinnitus.

^t^H-THI: Hearing-Tinnitus Handicap Inventory.

^u^RCI: Reliable Change Index.

^v^FU: follow-up moment.

^w^TS: Tinnitus Scale.

^x^NRS: Numeric Rating Scale.

^y^THI: Tinnitus Handicap Inventory.

^z^WN: white noise.

^aa^COSIT: Client-Oriented Scale of Improvement in Tinnitus.

^ab^SUS: System Usability Scale.

^ac^MAUQ: mHealth App Usability Questionnaire.

^ad^USL: UpSilent app.

^ae^BDI: Beck Depression Index.

^af^SSL: Subjective Stress Level.

^ag^RM: repeated measure.

^ah^NR: not reported.

^ai^STAI: State Trait Anxiety Inventory.

^aj^NS: not specified.

^ak^FTRS: Fudan Tinnitus Relieving System.

^al^HADS: Hospital Anxiety and Depression Scale.

^am^AIS: Acceptance of Illness Scale.

^an^FTQ: Fear of Tinnitus Questionnaire.

^ao^TCS: Tinnitus Catastrophizing Scale.

^ap^DCRS: digital customized relieving sound.

### Other Internet-Based Interventions

#### Study and Patient Characteristics

A total of 5 studies looked into the use of other internet-based interventions for chronic tinnitus, ranging from sound therapy, mindfulness, meditation, and intensive auditory cognitive training [24,28,29,31,47]. See Table S5 in Multimedia Appendix 4 for more information.

#### Results of Individual Studies

In total, 5 studies examined other internet-based interventions, not CBT-related [24,28,29,31,47]. All 5 studies looked into a different treatment, some more related to sound therapy, others focusing more on mindfulness or cognitive training. All studies reported promising results concerning the effectiveness of the treatment, except for Xing et al [47], who concluded that auditory intensive cognitive training was not associated with changes in self-reported tinnitus. Further details can be found in Table 5.

**Table 5:** Table of evidence for the studies concerning other internet-based interventions.

Study	Intervention					Outcome				Results				
Author and year	Intervention	Control group	Treatment period	Follow-up	Primary tinnitus outcome		Additional outcome measures	Statistical analysis method	Data results		Statistical analysis results	Dropout	Conclusion	GRADE^a^
Alonso-Valerdi et al. [24], 2021	(1) TRT^b^, (2) TEAE^c^, (3) ADT^d^, (4) BBT^e^, (5) music	No treatment	2 months (60 min daily)	FU1^f^: after 1 week; FU2^g^: after 5 weeks; FU3^h^: after 8 weeks	THI^i^		HADS^j^, audiometry, tinnitus characteristics analysis, and BL^k^ neurophysiological analysis.	Identification of ± or 0 effects *t* test.	All negative differences between BL and FU^l^ were statistically significant (*P*=.009).		29.4% of tinnitus sufferers in the TRT group reported diminution of their tinnitus perception, followed by those in TEAE (18.8%), BBT or ADT (14.3%), and music (7.7%) groups.	Dropout: music 6/16; BBT^m^ 4/18; TRT 3/18; TEAE 5/18; ADT 5/18; control 3/14.	TRT most effective sound-based therapy to reduce tinnitus perception for individuals without anxiety. BBT, ADT, and TEAE produced very similar effects.	⊕⊕⊕○
Connell et al [28], 2023	Customized acoustic therapy delivered through a web-based platform	N/A^n^	6 weeks (2 h daily)	Every week of treatment	FiveQ tinnitus symptoms severity scores		N/A.	Wilcoxon signed-rank tests, Mann-Whitney U-tests, linear mixed models, Tukey method *P* value adjustment for multiple variables, and a Kenward-Roger degrees of freedom estimation.	The mean difference between pre- and posttreatment of –9.3 (SD 15.5) was statistically significant (*P*<.001).		At treatment end point, 39/58 (67.2%) patients demonstrated an overall improvement in FiveQ, 4/58 (6.9%) had no change, and 15/58 (25.9%) declined.	51% (57/115) dropout.	The findings of this study support a customized acoustic therapy program as an efficacious tinnitus treatment modality that can improve symptom severity. Patients with low-frequency tinnitus and mild or above symptom severity at baseline demonstrated a greater treatment response.	⊕⊕⊕○
Gans et al [29], 2023	i-MBTSR^o^ focusing on applying mindfulness to tinnitus	N/A	8 weeks (2 h lesson/wk and 30 min daily instructor-led meditation practice)	FU1: posttreatment; FU2: 6 months posttreatment	TFI^p^		PSS^q^ TIQ^r^.	Repeated-measures 2 (group) × 3 (time) repeated-measures (using Wilks’ Lambda because of heterogeneity of variance) MANOVA^s^, ANOVA with the Greenhouse-Geisser method.	Post hoc contrast comparisons with Bonferroni adjustments were used to examine the differences between the levels of each main effect. Contrasts revealed that the greatest gains were made in TFI and PSS between time 1 and either time 2 or time 3 time periods.		31 (72%) patients experienced clinically meaningful improvements of 13 or more on the TFI.	534/577 (94%) did not complete FU1, and 555/577 (98%) FU2.	The iMBTSR course appears to be a viable and effective treatment modality for the tinnitus population.	⊕⊕⊕○
Jackson and Woolmer [31], 2024	Online body scan meditation intervention	Waiting list	8 weeks (3-4 times a week)	FU1: posttreatment; FU2: 6 months posttreatment	TFI		TCQ^t^ MAAS^u^.	1-way ANOVA design.	A significant main effect of condition on self-reported tinnitus distress measured by the TFI (*F*_1,100_=18.318; *P*<.001; 2*P*=.15) was present.		The mean TFI change over 8 weeks of body scan meditation was –9.52 in comparison to –0.58 for patients on the waiting list. 29.63% of the IG^v^ experienced a clinically significant change on the TFI of more than 14/100.	15/48 lost to follow-up in the control group and 9/54 in IG ≥LOCF^w^.	These results evidence that those in the body scan meditation group experienced a significant reduction in tinnitus distress, an increase in mindful thought, and a decrease in negative tinnitus cognitions.	⊕⊕⊕○
Xing et al [47], 2021	Brain HQ^x^ auditory intensive training	Brain HQ nonauditory intensive training	8 weeks (5 d/wk, 20 min/d)	FU1: posttreatment; FU2: 1 month posttreatment	TFI		TGBS^y^, CFQ^z^, BAI^aa^, PHQ-9^ab^.	Spearman correlation mixed effects model with an unrestricted variance-covariance matrix.	Marginal mean differences for IG and CG^ac^ were at 1 month (0.24, 95% CI –11.20 to 10.7), at 2 months 2.17 (95% CI –8.50 to 13.3), and at 3 months 3.36 (95% CI –7.91 to 14.6).		At 3 months, 13% of the CG and 19% of the IG experienced a clinically relevant reduction in the TFI of 13 or more.	>20%	Auditory intensive cognitive training was not associated with changes in self-reported tinnitus.	⊕⊕⊕○

^a^GRADE: Grading of Recommendations, Assessment, Development, and Evaluation.

^b^TRT: tinnitus retraining therapy.

^c^TEAE: therapy for an enriched acoustic environment.

^d^ADT: auditory discrimination therapy.

^e^BBT: Broad Band Therapy.

^f^FU1: first follow-up moment.

^g^FU2: second follow-up moment.

^h^FU3: third follow-up moment.

^i^THI: Tinnitus Handicap Inventory.

^j^HADS: Hospital Anxiety and Depression Scale.

^k^BL: baseline.

^l^FU: follow-up moment.

^m^BBT: broad band therapy.

^n^N/A: not applicable.

^o^iMBTSR: internet-based mindfulness-based tinnitus stress reduction.

^p^TFI: Tinnitus Functional Index.

^q^PSS: Perceived Stress Scale.

^r^TIQ: Tinnitus Impact Questionnaire.

^s^MANOVA: multivariate ANOVA.

^t^TCQ: Tinnitus Cognitions Questionnaire.

^u^MAAS: Mindful Attention Awareness Scale.

^v^IG: intervention group.

^w^LOCF: Last Observation Carried Forward.

^x^HQ: Hyperacusis Questionnaire.

^y^TGBS: Tinnitus Global Bothersome Scale.

^z^CFQ: Cognitive Failures Questionnaire.

^aa^BAI: Beck Anxiety Inventory.

^ab^PHQ-9: Patient Health Questionnaire-9.

^ac^CG: control group.

## Discussion

### Principal Findings

The previous systematic review published in 2023 on the topic of telerehabilitation for patients with chronic subjective tinnitus indicated that low-contact treatment provided from a distance through several telerehabilitation forms could be used as a substitution for or an addition to in-person clinical care for tinnitus [13]. It can potentially reduce the costs of treatment without sacrificing the level of effectiveness. Due to the advances in technology made every day, telerehabilitation is a quickly evolving research topic, and several new studies on telerehabilitation for tinnitus have been published in the last couple of years. Therefore, the aim of this systematic review was to give an updated overview of the research concerning the effectiveness of telerehabilitation interventions for self-management of tinnitus published between 2022 and 2025. The recent studies included in this review confirm the earlier findings that telerehabilitation shows promising results in effectively reducing tinnitus distress in a less costly and more accessible way. However, the recent findings also demonstrate some changes within the field of telerehabilitation for tinnitus.

In the last decade, a lot of research has been published concerning the development and effectiveness of iCBT programs. These studies formed the vast majority of the preceding review. In the last 3 years, a shift in telerehabilitation research has been noticeable. The interest in iCBT has abated, and the use of smartphone apps for telerehabilitation for tinnitus is a growing topic. Not only in the field of tinnitus, but in medicine and rehabilitation in general, the use of smartphone apps to offer treatment from a distance is on the rise and forms the newest trending topic in the field of medicine [49,50]. The implementation of smartphone apps offers several advantages over conventional internet-based interventions that typically rely on desktop or browser-based platforms. First, it ensures greater accessibility and reach, increasing the likelihood of consistent patient engagement and adherence. They also enable more flexible and context-sensitive delivery of rehabilitation tasks. Push notifications, reminders, and just-in-time prompts can support adherence and promote habitual use, increasing spontaneous use. Smartphone apps also tend to perform better in terms of user friendliness and patient satisfaction [51-53]. It is not just the use of apps, but also the content of the researched apps that has shifted. While earlier research investigated the use of apps specifically for sound therapy for tinnitus patients, the newer research also shows other treatments in the form of an app [54,55]. The majority of the researched apps still gravitate more toward the use of sound therapy, but also, CBT, TRT, and psycho-education are made more accessible in this way. It is, however, noticeable that while research concerning sound therapy in app form has scaled up its sample size in the last couple of years to strengthen its power, this is not yet the case for the other treatments in app form. CBT, TRT, and psycho-education without sound therapy were still investigated in smaller sample sizes and pilot studies. Additionally, 1 study looked into the use of an app with a cervical exercise program for patients with somatic tinnitus. These patients experienced neck complaints in addition to their tinnitus, and these complaints can influence the tinnitus sound and loudness due to somatosensory interference. As a tailored care approach detecting present influencing factors is recommended, it is good that telerehabilitation is expanding and not only focusing on the psychosocial aspect, but also on specific other potential factors that might be of influence, such as neck complaints.

Another trend noticeable from the research of the last couple of years is that internet-based programs are also expanding beyond iCBT with or without guidance. This review included studies looking into several forms of sound therapy, customized acoustic therapy, mindfulness, meditation, and even cognitive training through a web-based platform. These treatments all demonstrated promising results for reducing tinnitus distress. Except for the cognitive training program with acoustic cognitive exercises, which was not reported with self-reported changes in tinnitus experiences. However, given the promising results concerning the link between cognition and tinnitus and the potential use of cognitive training for tinnitus, future studies on cognitive training telerehabilitation forms for tinnitus remain relevant [56-58]. Sound therapy, meditation, and mindfulness have been widely researched in a nontelerehabilitation context for tinnitus, making it a great avenue to explore how these treatment forms can be further improved by increasing accessibility.

Important notes from the preceding review were the high dropout rates from iCBT programs and the lack of reporting dropout in studies concerning the use of smartphone apps [13]. The same note can still be made in the current review. For iCBT, 2 of 5 studies reported dropout, which was around 34% at postintervention follow-up. This is in line with previous similar studies concerning iCBT in this population. According to Beukes et al [26], patients withdraw from treatment due to time constraints or lack of engagement. In accordance, the preceding review stated that earlier research indicated that barriers such as lack of time, engagement, motivation, and openness of the patient cause high dropout and should be taken into consideration. The previous review also concluded that future research should further explore which factors are most likely to cause the lack of compliance and how clinicians can counteract these. Therefore, it is crucial that studies analyze and report where the dropout is coming from, as Beukes et al [26] did extensively in their discussion. For the smartphone apps, a change could be noted, namely, the dropout rates were reported in 8 of 13 studies and ranged from no dropout to 53%. In other self-management intervention forms discussed, the rate ranged up to 94%, but on average was between 34% and 63%. The reasons for dropout noted were related to practical obstacles or treatment preferences from the patients. Oron et al [36] offered that monitoring programs might enhance the retention of treatment. In other treatment forms, the dropout rate and reasons for dropout were often not listed. As stated in the preceding review, to enhance the effectiveness of telerehabilitation, the limited current understanding of predictors of dropout should be tackled. Additionally, potential ways to improve adherence, such as therapist guidance levels, motivational feedback, automated reminders, etc, should be explored further [59].

### Limitations

The included studies of this systematic review presented similar limitations as earlier research concerning this topic summarized in the previous review. All included studies scored some to great concerns of RoB. As a result, it needs to be acknowledged that the overall certainty of evidence ranged from low to moderate certainty. In addition, some crucial confounding parameters, such as the presence of hearing loss, hyperacusis, anxiety, depression, or sleeping problems, were not taken into consideration by all studies. However, it needs to be noted that concerning the presence of hearing loss, improvements were made on this level. A total of 15 of 24 studies reported how large the proportion of participants with hearing loss was or how many patients used hearing aids. Yet, how they defined the presence of hearing loss was often not clarified. An audiometric examination of tinnitus patients is therefore of value when researching patients with tinnitus because hearing loss is an important risk factor for developing tinnitus [1]. Due to the additional distress of hearing loss, tinnitus patients with hearing loss might present with worse tinnitus distress levels in comparison to tinnitus patients without hearing loss, according to earlier research [60].

While hearing loss is an important confounding factor to keep in mind, other factors also need to be considered: gender, age, mental state, hyperacusis, and neck and jaw complaints [61-71]. These factors might play a role in how patients perceive their tinnitus and how they react to treatment forms [61-71]. Additionally, the link between tinnitus and comorbidities such as anxiety, depression, stress, sleeping problems, hyperacusis, etc, are already widely researched [66,72-74]. The more serious these comorbidities are, the worse the prognosis of the tinnitus [73]. All the factors mentioned above might have an impact on the effectiveness of a treatment and should, therefore, be considered in future research. Additionally, for this reason, there is an increasing need for personalization of the treatment approach. Currently, often 1 factor is taken into consideration; however, a multidisciplinary and tailored approach is recommended. Therefore, the generalizability of these results is low, tinnitus mechanisms can greatly differ individually, and the limited tailoring options of these self-management interventions cause the evidence concerning the efficacy to remain rather limited. Due to a lack of high-quality and homogeneous studies concerning this topic, no confident conclusions can be made concerning the generalizability of self-management interventions for patients with tinnitus. Telerehabilitation has the potential to make treatment tailored to the needs of the patients more accessible; however, future research should invest more into using this potential. Future studies should focus on delivering high-quality study designs with homogenous outcomes to build the evidence of the strengths of telerehabilitation, which are now considered potential benefits.

### Conclusions

This updated systematic review concerning the effectiveness of telerehabilitation for patients with chronic subjective tinnitus confirms the conclusion stated in the preceding review. The results of this systematic review indicate that telerehabilitation in the form of iCBT with guidance, self-help manuals, self-help devices, smartphone apps, and other internet-based interventions (except for cognitive training) are effective in relieving tinnitus distress. Telerehabilitation might form an alternative or additional tool to the recommended in-person care that patients currently receive. This review accentuated the shift from iCBT to the growing interest in the use of smartphone apps, increasing the accessibility of the treatments even more. However, the most significant barrier to the success of telerehabilitation remains the lack of compliance with the treatment. The great expansion of research within the field of telerehabilitation for tinnitus treatment in recent years indicates that it remains an evolving topic to keep track of.

## Appendix

Multimedia Appendix 1 PRISMA checklist for systematic review.

Multimedia Appendix 2 Search strategy updated.

Multimedia Appendix 3 Risk of bias summarization of newly included papers.

Multimedia Appendix 4 Tables of study and patient characteristics of newly included papers.

## Abbreviations

CBT: cognitive behavioral therapy
CG: control group
GRADE: Grading of Recommendations, Assessment, Development, and Evaluation
iCBT: internet-based cognitive behavioral therapy
MeSH: Medical Subject Headings
PICO: population, intervention, comparison, and outcome
PRISMA: Preferred Reporting Items for Systematic Reviews and Meta-Analysis
PRISMA-S: Preferred Reporting Items for Systematic Reviews and Meta-Analyses Literature Search Extension
RCT: randomized controlled trial
RoB: risk of bias
ROBINS-I: Risk of Bias in Non-Randomized Studies of Interventions
TFI: Tinnitus Functional Index
TRT: tinnitus retraining therapy

## Data Availability

All data underlying the findings of this systematic review are derived from the studies included in the review and are publicly available through the original publications, which are fully referenced in this paper. No additional primary data were generated or collected for this work. Extracted datasets and materials used for the review process are available from the corresponding author upon reasonable request.
