# Supplementary material for: Effectiveness of Telerehabilitation Interventions for Self-Management of Tinnitus: Update of a Systematic Review
Source: J Med Internet Res. 2026 Feb 27;28:e83529. doi: 10.2196/83529 (PMC12988352; doi:10.2196/83529)
Supplement: Multimedia Appendix 2 [file jmir_v28i1e83529_app2.doc]

| **Database** | **Search string** | **articles** |
| --- | --- | --- |
| **Pubmed** | *Tinnitus [MeSH Terms] AND (("internet based intervention"[MeSH Terms]) OR (self-care[MeSH Terms]) OR (self-management[MeSH Terms]) OR ("mobile applications"[MeSH Terms]) OR ("telerehabilitation"[MeSH Terms]) OR (smartphone[MeSH Terms]) OR (Telemedicine [MeSH Terms]) OR Telehealth OR Telemedicine OR smartphone OR telerehab* OR “mobile application” OR “self manag*” OR “self-care” OR “internet based intervention")* | 74  118  44 |
| **Web of science** | TS=(self NEAR/3 (care OR management OR help) OR mobile applications OR internet based intervention OR web based intervention OR online intervention OR internet intervention OR telerehabilitation OR smartphone OR telemedicine OR telehealth OR mobile apps OR software apps OR virtual rehabilitation) AND TS=(tinnitus) | 228  345  117 |
| **Science Direct** | Tinnitus AND Smartphone application | 51  147  96 |
| **Cochrane Library** | *("self-care" OR "self-care" OR "self-management" OR "self-management" OR "self-help" OR "self-help" OR "mobile applications" OR "internet based intervention" OR "internet-based intervention" OR "web based intervention" OR "web-based intervention" OR "online intervention" OR "internet intervention" OR "telerehabilitation" OR "tele-rehabilitation" OR smartphone OR telemedicine or tele-medicine OR telehealth OR tele-health OR "mobile apps" OR "software apps" OR "virtual rehabilitation") AND (tinnitus) in All Text | 56  95  39 |
| **Scopus** | ALL ( self  W/3  ( care  OR  management  OR  help )  OR  ( "mobile applications" )  OR  ( "internet-based intervention" )  OR  ( "web-based intervention" )  OR  ( "online intervention" )  OR  ( "internet intervention" )  OR  telerehabilitation  OR  telemedicine  OR  ( "mobile apps" )  OR  ( "software apps" )  OR  ( "virtual rehabilitation" ) )  AND  ALL ( tinnitus ) | 146  487  341 |
| **Total** | | 555  1192  637 |

Update 10/02/2025

New screened articles
