# Supplementary figures and images for: Effectiveness of Telerehabilitation Interventions for Self-Management of Tinnitus: Update of a Systematic Review
Source: J Med Internet Res. 2026 Feb 27;28:e83529. doi: 10.2196/83529 (PMC12988352; doi:10.2196/83529)

**Figure S1.** Risk of bias controlled trials.


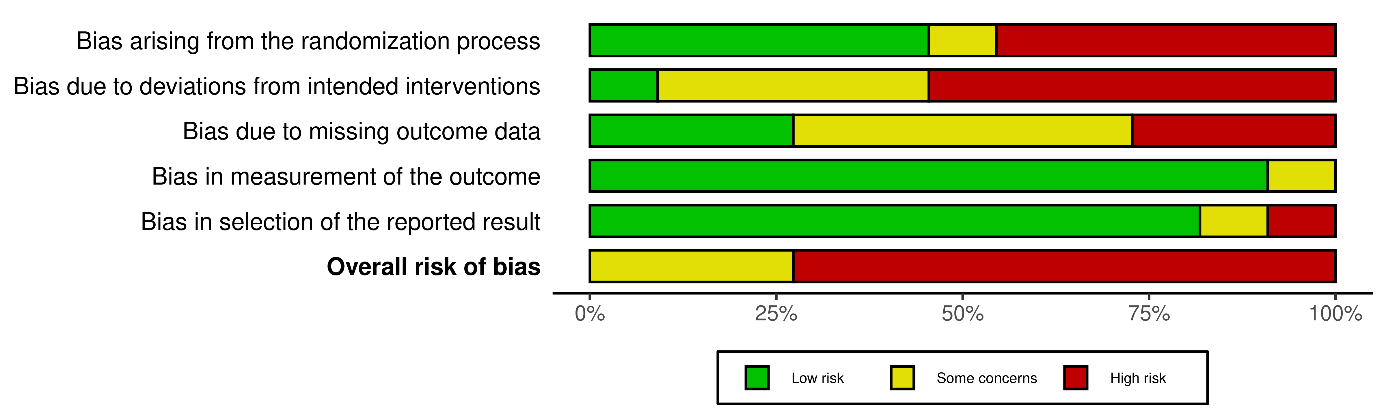


**Figure S2.** Risk of bias noncontrolled trials.


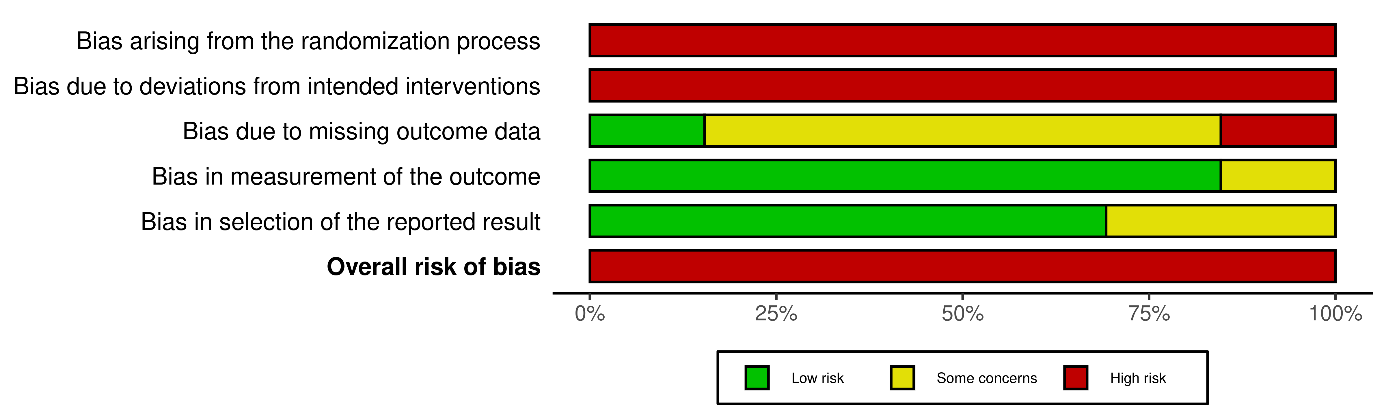

Supplement: Multimedia Appendix 3 [file jmir_v28i1e83529_app3.docx]
