# Supplementary material for: Effectiveness of Telerehabilitation Interventions for Self-Management of Tinnitus: Update of a Systematic Review
Source: J Med Internet Res. 2026 Feb 27;28:e83529. doi: 10.2196/83529 (PMC12988352; doi:10.2196/83529)
Supplement: Multimedia Appendix 4 [file jmir_v28i1e83529_app4.docx]

| **Table S1.** Study and population characteristics iCBT with guidance. | | | | | | | | | | | |
| --- | --- | --- | --- | --- | --- | --- | --- | --- | --- | --- | --- |
| **Study characteristics** | | | | | **Population characteristics** | | | | | | |
| **Author** | **Date** | **Country** | **Design** | **Category** | **Sample size** | **Gender (%)** | **Mean age (SD) (years)** | **Type of tinnitus** | **mean duration of tinnitus (SD) (years)** | **Tinnitus characteristics** | **Presence of hearing loss (%)** |
| **Beukes**  **et al.** | 2022 | USA | CH |  | n=27 | ♂: 33% ♀: 67% | 55,48 (+/-9,9) [34-71] | Chronic tinnitus (>3 months) with a score of more than 25/100 on the TFI | 11,75 (+/- 13,36) | NS | Hearing aid use Bilateral: 7 (26%) Unilateral: 3 (11%) Hearing aids help mask the tinnitus: 4 (40%) Hearing aids don’t mask the tinnitus: 6 (60%) |
| **Beukes**  **et al. (2nd)** | 2022 | USA | CH |  | n=200 | ♂: 44% ♀: 56% | 56,33 (+/-12,48) [22-84] | Chronic tinnitus | 11,64 (+/-14,57)[0,3-70] | Tinnitus location: Right: 16% Left: 13% Both ears: 57% In the head: 14% | NS |
| **Manchaiah et al.** | 2024 | USA | CH |  | n=164 | ♂: 41% ♀: 59% | 58,44 (+/- 11,45) | Chronic subjective tinnitus (>3 months) with at least a mild severity of tinnitus | 13,91 | NS | NS |
| **Rodrigo et al.** | 2021 | USA | CH |  | n=228 | ♂: 57% ♀: 43% | 55,14 (+/-12,92) | long standing chronic tinnitus | 17,68 (+/-19,42) | 38,5% unilateral 61,5% bilateral | 30,3% wore hearing aids |
| **Rodrigo et al. (2nd)** | 2021 | USA | CH |  | n=228 | ♂: 57% ♀: 43% | 55,14 (+/-12,92) | long standing chronic tinnitus | 17,68 (+/-19,42) | 38,5% unilateral 61,5% bilateral | 30,3% wore hearing aids |

USA= United States of America; CH= Cohort study; n= sample size; TFI= Tinnitus Functional Index; NS= Not specified

| **Table S2.** Study and population characteristics self-help devices***.*** | | | | | | | | | | | |
| --- | --- | --- | --- | --- | --- | --- | --- | --- | --- | --- | --- |
| **Study characteristics** | | | | | **Population characteristics** | | | | | | |
| **Author** | **Date** | **Country** | **Design** | **Category** | **Sample size** | **Gender (%)** | **Mean age (SD) (years)** | **Type of tinnitus** | **mean duration of tinnitus (SD) (years)** | **Tinnitus characteristics** | **Presence of hearing loss (%)** |
| **Alonso-Valerdi et al.** | 2021 | Mexico | CT |  | n=108 CG: n=18 IG: n=90 | CG: ♂: 61% ♀: 39% IG: ♂: 41% ♀: 59% | CG: [22-70] IG:  [37-80] | NS | NS | tinnitus was mostly perceived at 8 kHz (29%) with an intensity between 31 and 40 dB (26%) on the left side (44%) | 32% had no hearing loss ( PTA< 20dB) |
| **Altissimi et al.** | 2024 | Italy | RCT |  | n=80 | ♂: 60% ♀: 40% | 46,1 | Continuous Subjective tinnitus | 0-6months: 22,5% 7-12months: 25% 1-3 years: 40% 3-5years: 7,5% >5years: 5% | 35% unilateral 52,5% bilateral 12,5% undefined | normal hearing to mild hearing loss without the use of hearing aids |
| **Searchfield et al.** | 2022 | Australia | RCT |  | n=98 CG: n=48 IG: n=50 | CG: ♂: 61% ♀: 39% IG: ♂: 41% ♀: 59% | 53 (+/-15) | Chronic moderate- severe tinnitus (>6 months+ > 40/100 on TFI) | 15 (+/- 18) | Tinnitus location: Right: 10% Left: 16% Left of center: 13% Right of center: 13% Equal ears: 32% In head: 16% | 22,6% had hearing aids |

CT= Control Trial; n= sample size; CG= control Group; IG= Intervention Group; NS= Not specified; PTA= Pure Tone Audiometry; RCT= Randomised Controlled Trial

| **Table S3.** Study and population characteristics self-help manual. | | | | | | | | | | | |
| --- | --- | --- | --- | --- | --- | --- | --- | --- | --- | --- | --- |
| **Study characteristics** | | | | | **Population characteristics** | | | | | | |
| **Author** | **Date** | **Country** | **Design** | **Category** | **Sample size** | **Gender (%)** | **Mean age (SD) (years)** | **Type of tinnitus** | **mean duration of tinnitus (SD) (years)** | **Tinnitus characteristics** | **Presence of hearing loss (%)** |
| **Shetty et al.** | 2024 | India | CH |  | n=10 | ♂: 80% ♀: 20% | 35,4 [20-45] | NS | NS | NS | patients with bilateral mild sloping sensoneural hearing loss who wear a hearing aid |

CH= Cohort study; n= sample size; NS= Not Specified

| **Table S4.** Study and population characteristics smartphone applications. | | | | | | | | | | | |
| --- | --- | --- | --- | --- | --- | --- | --- | --- | --- | --- | --- |
| **Study characteristics** | | | | | **Population characteristics** | | | | | | |
| **Author** | **Date** | **Country** | **Design** | **Category** | **Sample size** | **Gender (%)** | **Mean age (SD) (years)** | **Type of tinnitus** | **mean duration of tinnitus (SD) (years)** | **Tinnitus charact-eristics** | **Presence of hearing loss (%)** |
| **Altissimi et al.** | 2024 | Italy | RCT |  | n=80 | ♂: 60% ♀: 40% | 46,1 | Continous Subjective tinnitus | 0-6  months: 22,5% 7-12months: 25% 1-3 years: 40% 3-5years: 7,5% >5years: 5% | 35%  unilateral 52,5% bilateral 12,5% undefined | normal hearing to mild hearing loss without the use of hearing aids |
| **Goshtasbi et al.** | 2025 | USA | RCT |  | n=92 CG: n=45 IG: n=47 | CG: ♂: 60% ♀: 40% IG: ♂: 66% ♀: 34% | CG: 58,1 (+/-11,6) IG: 56,3 (+/-12,1) | constant moderate-to-severe tinnitus, characterized by a baseline TFI ranging from 18-65 (moderate) or >65 (severe) for at least 6 months | CG:  8,0 (+/-7,4) IG:  7,2 (+/-9,1) | CG: 17,8% unilateral 26,7% bilateral equally 55,6% bilateral unequally IG: 12,8% unilateral 27,7% bilateral equally 59,6% bilateral unequally | NS |
| **Kutyba et al.** | 2022 | Poland | CH |  | n=52 | ♂: 48% ♀: 52% | 48 (+/- 13,8) [18-73] | Chronic tinnitus | 5,4 (+/-5,9) [1-30] | 38,5% unilateral 61,5% bilateral | PTA AC R/L: 20,0 dB (+/-12,7)/ 20,8 dB (+/-11,8) PTA BC R/L: 16,0 dB (+/- 12,3)/ 16,3 dB (+/-11,5) |
| **Kutyba et al. (2nd)** | 2022 | Poland | CT |  | (originally enrolled n=147) but included for analysis : n=68 CG: n=24 IG: n=44 | CG: ♂: 46% ♀: 54% IG: ♂: 43% ♀: 57% | CG: [28-74]; 51,9 (+/-14,1) IG: [26-72];51,9 (+/-11,6) | Chronic tinnitus (>6 months) | CG: [0,7-40]; 6,2 (+/-8,1) IG: [1-26]; 4,7 (+/-5,4) | CG: 50% unilateral 50% bilateral 92% constant 8% intermittent IG: 30% unilateral 70% bilateral 93% constant 7% intermittent | CG: 54% had hearing loss IG: 50% had hearing loss |
| **Michiels et al.** | 2024 | Belgium | RCT |  | (assessed for eligibility: n=138) but included for analysis : n=38 CG: n=19 (3 dropt-outs) IG: n=19 (4 drop-outs) | CG: ♂: 56% ♀: 44% IG: ♂: 40% ♀: 60% | CG: 50,93 (+/-12,78) IG: 48,00 (+/-13,60) |  | CG: 7,9 (+/-7,88) IG: 7,5 (+/-7,06) | NS | NS |
| **Oron et al.** | 2022 | Israel | CH |  | (originally enrolled n=26) but included for analysis : n=14 | ♂: 57% ♀: 43% | 51,9 (+/- 15,3) | constant (subjective and non-pulsatile) tinnitus lasting more than 3 months with vertigo complaints | 7,5 (+/- 8,3) | NS | 9 patients with sensorineural hearing loss; 2 patients with mixed hearing loss; 2 patients with conductive hearing loss; 1 patient with normal hearing |
| **Ravi et al.** | 2025 |  | CT |  | n=2 | Patient A= male; Patient B= female | Patient A= 35 Patient B= 38 | ideopathic tinnitus | 3months for both patients | NS | Patient A= bilateral minimal hearing loss; Patient B= bilateral hearing sensitivity within normal limits |
| **Schlee et al.** | 2022 | Germany | CH |  | (originally enrolled n=62) but included for analysis : n=36 | ♂: 61,1% ♀: 38,9% | 49,4 (+/-11,7) | Chronic tinnitus | 8,6 (+/- 17,5) | NS | NS |
| **Searchfield et al.** | 2022 | Australia | RCT |  | n=98 CG: n=48 IG: n=50 | CG: ♂: 61% ♀: 39% IG: ♂: 41% ♀: 59% | 53 (+/-15) | Chronic moderate- severe tinnitus (>6 months+ > 40/100 on TFI) | 15 (+/- 18) | Tinnitus location: Right: 10% Left: 16% Left of center: 13% Right of center: 13% Equal ears: 32% In head: 16% | 22,6% had hearing aids |
| **Seol et al.** | 2023 | Republic of Korea | CH |  | n=22 | ♂: 77% ♀: 23% | 52,2 (+/-13,4) [24-71] | subjective and chronic tinnitus (≥3 months) | 6,2 (+/- 5,9) | The number of ears with tinnitus was 20 for the right ear and 16 for the left ear | Mean PT thresholds: right ear: 24,4 (+/-15,2)dB left ear: 26,9 (+/- 17,2) dB |
| **Suh et al.** | 2023 | Republic of Korea | CT |  | n=84 CG: n=42 IG: n=42 | ♂: 48% ♀: 52% | 57,9 ( +/-11,1) | Chronic tinnitus (>6 months) | NS | Tinnitus location: Right: 23% Left: 33% Both ears: 32% In head or unclear: 12% | Mean PT thresholds: right ear: CG: 25,6 (+/-14,6)dB IG: 23,2 (+/-14,6)dB left ear: CG:24,0 (+/-15,4) dB IG: 28,9(+/-15,4)dB |
| **Tang et al.** | 2022 | China | CH |  | n= 22867 | ♂: 60% ♀: 40% | ♂: 34 (IQR= 27-43) ♀: 36 (IQR=28-48) | NS | ♂: 9 months; (IQR=2-36) ♀: 6 months ; (IQR= 2-24) | overview table for men and women for count of tinnitus sound types, dominant matched pitch, position cerebri | NS |
| **Tang et al. (2nd)** | 2025 | China | CT |  | n=184 CG: n=107 IG: n=77 | CG: ♂: 50,5% ♀: 49,5% IG: ♂: 55% ♀: 45% | CG: 45 (IQR=35,5-57) IG: 50 (IQR= 37-59) | Subjective tinnitus | CG: 12 months (IQR=4-24) IG: 8 months (IQR=3-42) | CG: 48% unilateral 4% bilateral 48% in head IG: 37% unilateral 7% bilateral 56% in head | Median PT thresholds: right ear: CG: 16,67 (IQR= 15-20,42)dB IG: 16,67 (IQR= 15-21,67)dB left ear: CG: 17,50 (IQR= 15-23,75) dB IG: 16,67 (IQR= 15-23,33)dB |

RCT= Randomised Controlled Trial; n= sample size; CG= Control Group; IG= Intervention Group; CH= Cohort Study; CT= Controlled Trial; PTA= Pure Tone Audiometry; PT= Pure Tone; AC= Air Conduction; BC= Bone conduction; dB= decibel; R= Right; L= Left; NS= Not specified; IQR= InterQuartile Range

| **Table S5.** Study and population characteristics other internet-based interventions. | | | | | | | | | | | |
| --- | --- | --- | --- | --- | --- | --- | --- | --- | --- | --- | --- |
| **Study characteristics** | | | | | **Population characteristics** | | | | | | |
| **Author** | **Date** | **Country** | **Design** | **Category** | **Sample size** | **Gender (%)** | **Mean age (SD) (years)** | **Type of tinnitus** | **mean duration of tinnitus (SD) (years)** | **Tinnitus characteristics** | **Presence of hearing loss (%)** |
| **Alonso-Valerdi et al.** | 2021 | Mexico | CT |  | n=108 CG: n=18 IG: n=90 | CG: ♂: 61% ♀: 39% IG: ♂: 41% ♀: 59% | CG: [22-70] IG: [37-80] | NS | NS | tinnitus was mostly perceived at 8 kHz (29%) with an intensity between 31 and 40 dB (26%) on the left side (44%) | 32% had no hearing loss ( PTA< 20dB) |
| **Connell et al.** | 2023 | Australia | CH |  | n=58 | ♂: 74,1% ♀: 25,9% | 25-34 yrs: 3,4% 45-54 yrs: 17,2% 55-64: 43,1% 65-74: 32,6% >75: 3,4% | Subjective non-pulsatile tinnitus | 0-4 yrs: 17,2% 5-9 yrs: 13,8% 10-14 yrs: 20,7% 15-19 yrs: 12,1% 20+ yrs: 36,2% | 15,5% unilateral 84,5% bilateral 55,2% single tone 44,8% multitone | 32,6% had hearing loss 24,1% wore hearing aids |
| **Gans et al.** | 2023 | USA | CH |  | n=43 | ♂: 51,2% ♀: 48,8% | NS | adult patients with moderate to severe tinnitus distress according to the TFI (TFI>25) | NS | NS | NS |
| **Jackson et al.** | 2024 | UK | RCT |  | n=108 CG: n=48 IG: n=54 | CG: ♂: 52% ♀: 48% IG: ♂: 38% ♀: 62% | CG: 55,85 (+/-11,76) IG: 51,33 (+/-13,21) | Chronic tinnitus (>6 months) | CG: 9,4 (+/-8,56) IG: 8,76 (+/-8,66) | NS | NS |
| **Xing et al.** | 2021 | USA | RCT |  | n=125 CG: n=63 IG: n=62 | CG: ♂: 53% ♀: 47% IG: ♂: 72% ♀: 28% | CG: 61 [34-68] IG: 63 [25-69] | subjective idiopathic nonpulsatile tinnitus with a Tinnitus Global Bothersome Scale score 3 (on a 1-5 scale, with 5 being the worst). | CG: [0-28,5]; 4,3 IG: [0-47,6]; 6,2 | NS | NS |

CT=Controlled Trial; n= sample size; CG= Control Group; IG= Intervention Group; NS= Not Specified; PTA= Pure Tone Audiometry; CH= Cohort; USA= United States of America; TFI= Tinnitus Functional Index; UK= United Kingdom; RCT= Randomised Controlled Trial
